# Sustainable Earnings among Immigrants, and the Role of Health Status for Self-Sufficiency: A 10-Year Follow-Up Study of Labour Immigrants and Refugees to Sweden 2000–2006

**DOI:** 10.3390/ijerph20010663

**Published:** 2022-12-30

**Authors:** Magnus Helgesson, Maria Brendler-Lindqvist, Bo Johansson, Tobias Nordquist, Martin Tondel, Magnus Svartengren

**Affiliations:** 1 Department of Medical Sciences, Occupational and Environmental Medicine, Uppsala University, SE-752 37 Uppsala, Sweden; 2 Department of Public Health and Caring Sciences, Health Equity and Working Life, Uppsala University, SE-752 37 Uppsala, Sweden; 3 Occupational and Environmental Medicine, Uppsala University Hospital, SE-751 85 Uppsala, Sweden

**Keywords:** self-sufficiency, refugees, immigrants

## Abstract

This study aimed to investigate economic self-sufficiency for immigrants, and how health status affected self-sufficiency. The proportion of self-sufficiency during years 1–10 after receiving a residence permit is presented for all non-European labour immigrants (*n* = 1259) and refugees (*n* = 23,859), aged 18–54, who immigrated to Sweden 2000–2006, and compared to a control group of Swedish-born (*n* = 144,745). The risk of not being self-sufficient in year 10 was analysed with Cox regression models, and the results are presented as hazard ratios (HRs) with 95% confidence intervals (CIs). Moreover, the impact on the self-sufficiency of having a diagnosis from specialised health care during the first five years in Sweden was analysed. The results showed that half of the refugees and three-quarters of the labour immigrants were self-sufficient 10 years after residency. The adjusted risk of not being self-sufficient at year 10 was 80% higher among labour immigrants (HR = 1.8; CI = 1.6–2.0) and more than two-fold among refugees (HR = 2.7; CI = 2.6–2.8) compared to the Swedish-born. Having a diagnosis from specialised health care during the first five years in Sweden had an impact on self-sufficiency in all groups; however, the impact of having a diagnosis did not differ between refugees and Swedish-born. Measures must be taken to increase immigrants’ work participation.

## 1. Background

Immigrants make important contributions to the labour force in Western countries, but labour market integration of immigrants also brings challenges [1]. Sweden is a case in point as it is one of the European countries with the highest reception of refugees per capita. Since the beginning of the 2000s, Sweden has received more than one million immigrants, with peaks of 100,000 to 150,000 annual refugees from ongoing wars in Syria, Afghanistan and recently, Ukraine. Currently, one-fifth of the total population of around 10 million inhabitants are persons born outside Sweden [2]. Until the 1970s, predominantly, labour immigrants from the Nordic countries and southern Europe immigrated to Sweden. Changes in the Swedish immigration regulations at the beginning of the 1970s significantly decreased labour immigration. Instead, mostly refugees from outside Europe arrived in Sweden [3]. Being self-sufficient by having employment has been the top priority for the Swedish integration policy since the Second World War, and political measures have been implemented to facilitate immigrants’ possibilities to establish themselves in the labour market [4]. Despite all these efforts, immigrants have a substantially higher unemployment rate compared to individuals born in Sweden [5]. Some regulations make it difficult for asylum seekers to seek work before a residence permit is granted. Not only can this period before finding out if the residence permit has been granted be very long for many individuals, but it can also be quite challenging [6]. Furthermore, once an individual receives a residence permit, the time until one is established in the labour market could be long. One shall also bear in mind that even after first entry into the labour market, immigrants face a much higher risk of entering unemployment compared to the host population [1]. Studies from Australia and Canada report that immigrants have difficulties in establishing themselves in the labour market [7,8,9]. In addition, studies from Germany and Turkey, which besides Sweden have welcomed the most refugees per capita in Europe, show that refugees are struggling to get a foothold in the labour market [10,11]. Several factors have been identified as obstacles to entering the labour market, including a low educational level, language barriers and poor social networks. Discrimination might be another barrier to overcome [12,13]. However, most existing studies are qualitative and evaluate interventions to enhance economic integration rather than empirically assessing the time until economic independence. Thus, very few studies have in a population-based setting assessed the time until immigrants reach a reasonable income level in the host country. One study from Sweden reports for instance that around 30% of non-labour migrants who came to Sweden between 1990 and 2014 had stable employment after three years in Sweden, and that it took up to 15 years for 80% of a cohort to complete labour market entry [14]. Another Swedish study showed that a duration of residence >10 years positively influenced the risk of long-term unemployment among African and Asian immigrants to Sweden [15]. It is also common that immigrants who are in employment have jobs with unfavourable physical and psychosocial work conditions. In addition, immigrants more often have insecure employment contracts and lower pay compared to people born in Sweden [14,15,16]. Further, one’s civil status and children living at home have been reported to influence the ability to work; being married and having children at home may decrease the ability to have paid work [17]. All these factors have been reported to increase the risk of periods with long-term sick leave and the granting of a disability pension [15,18]. Moreover, unemployment and long periods outside the labour market tend to be a vicious circle, as inactivity further aggravates both the deterioration of health and the possibility of finding employment [16,19]. 

The role of immigrants’ health in difficulties in becoming established in the labour market is not much studied. In studies of the general immigrant populations, immigrants often present better health and lower mortality compared to their counterparts remaining in their home country, and in some cases also compared to the general population in the host country (healthy migrant effect) [20,21]. However, this may primarily apply to labour immigrants. For refugees, events before and during flight may impact their health negatively, and studies have reported that refugees are more susceptible to mental disorders compared to the host population [22]. In Sweden, newly arrived immigrants present better physical health compared to Swedish-born, while the opposite is true for mental health [23]. Results from a survey on the health of newly arrived refugees in Skåne County presented similar results; however, about 20% of respondents in this study reported health problems to be a barrier to their job-seeking activities [24].

### Aims

Our study aimed to investigate economic self-sufficiency for labour immigrants and refugees during the first ten years after receiving a residence permit in Sweden compared to a control group of Swedish-born. The study also aimed to investigate how the self-sufficiency of labour immigrants, refugees and Swedish-born was affected by their health status during the first five years in Sweden.

## 2. Methods

### 2.1. Study Population

The study base included annual cohorts of all categorised labour immigrants and refugees to Sweden from outside Europe, who received a residence permit from 2000 to 2006 and were aged from 18 to 54 at this time (*n* = 87,681). In our study, an “immigrant” was defined as a person born outside Europe with two parents born outside Sweden, and a “Swedish-born” was defined as a person born in Sweden with two parents born in Sweden. Based on classifications by the Swedish Migration Agency, at the time of being granted a residence permit, the immigrants could be differentiated into labour immigrants and refugees. We chose to focus on non-European immigrants, as they are the immigrants who have been reported to face most challenges in the labour market, with considerably higher unemployment rates compared to European immigrants [15]. The annual cohorts were defined as the 31st of December each year. Since data regarding the reason for a residence permit are lacking for many individuals before the year 2008, 2141 individuals with missing data or no specified information on this variable were excluded from the study. Moreover, individuals who immigrated for reasons other than labour or as asylum seekers were excluded, such as students (*n* = 6712) and family reunification immigrants (*n* = 48,709), because the registers, for example, do not differentiate between the reunion of labour immigrants and refugees before the year 2011. As we were examining sustainability in the labour market, we had to restrict our study population to only those individuals with information in national registers for 10 consecutive years after receiving a residence permit; from the cohort of 30,119 individuals, 4691 individuals that emigrated from Sweden within 10 years after arrival were excluded. Furthermore, individuals that died (*n* = 230) or had missing data in registers (*n* = 80) were excluded. The final cohort included a total of 25,118 immigrants (approximately 29% of the study base), including 1259 labour immigrants and 23,859 refugees. The year when the residence permit was granted was labelled year 0 for all groups, and the follow-up period stretched from year 1 to year 10, with information on annual income from work and health status. The immigrants were compared to a five-times-bigger control group of Swedish-born individuals with the same age and sex composition as the study base of the immigrants. The Swedish-born were also given a fictive “year of residence” that corresponded in numbers to the year of residence of the immigrants of the study base. As a higher proportion of immigrants than Swedish-born were not listed in the register for 10 consecutive years, the study population ended up with 144,745 Swedish-born controls.

### 2.2. Registers and Data

Demographic and socioeconomic data were collected from four national registers held by Statistics Sweden: the Longitudinal Integration Database for Health Insurance and Labour Market Studies (LISA), the Longitudinal Database for Integration Studies (STATIV), Register-based labour market statistics (RAKS), and The Register over the Total Population (RTB); data on demographic and socioeconomic variables, sex, age, civil status and children living at home (measured at the year when residence permit was received), educational level (measured at year 10 due to a large number of missing data at year 1) and annual income from work were collected from the LISA and RAKS databases. Information on the immigrants’ reason for seeking a residence permit (labour immigrant or refugee) was obtained from the STATIV and RTB databases. Data on health status were measured as a diagnosis of either somatic or psychiatric health care and were retrieved from the National Patient Register (NPR) held by the National Board of Health and Welfare.

### 2.3. Definitions of Important Variables

The price base amount (PBA) is an annually updated index variable by Statistics Sweden, which reflects the actual price level in Sweden, covering general inflation [25]. An annual income of 3.5 PBA has been regarded as the minimum level where a person is economically self-sufficient [26]. In our study, we chose to make a cut-off for annual income from work at half that amount, 1.75 PBA, which means that the person has at least half of what is needed for economic self-sufficiency, and thereby indicates a foothold in the labour market. In this study, the variable self-sufficiency was defined as an income from paid work that equalled or exceeded 1.75 times the actual PBA. The PBA was approximately EUR 3700 in 2001, and it followed the annual inflation and increased to approximately EUR 4400 in 2016. 

### 2.4. Health Status

The NPR includes information about the main diagnosis at discharge from hospitalisation and specialised outpatient physician visits, coded according to the International Classification of Diseases, version 10 (ICD-10). Public and private caregivers must report to the register. To study the impact of health status on self-sufficiency, those individuals who were hospitalised or visited a physician within specialised outpatient care due to any of the included diagnoses at least once (first diagnosis within the first five years since residency or fictive year of residency for the Swedish-born) were compared with individuals who did not receive any of these diagnoses during the same period. The included diagnoses were from the ICD-10 chapters Mental and behavioural disorders, Diseases of the circulatory system, Diseases of the respiratory system or Diseases of the musculoskeletal system and connective tissue. They represented approximately 83% of all diagnoses from hospitalisation and specialised outpatient physician visits within the study population during years 1–5 after receiving a residence permit. We chose to include chronic diseases or diseases with a high risk of relapse and thereby with an assumed effect on work capacity. Using individual identification numbers for all people in Sweden, the demographic, socioeconomic and health information could be linked to each individual and subsequently be provided to us with a serial de-personalised number by Statistics Sweden. All data were thus coded to protect the personal integrity of the study population. 

### 2.5. Statistical Analyses

Descriptive statistics were used to present the proportion of self-sufficiency during years 1 to 10 among labour immigrants, refugees and Swedish-born, stratified by sex. Separate figures are presented for individuals with and without employment in year five, as well as for individuals with and without a diagnosis during years 1–5. 

Cox regression models computed hazard ratios (HRs) with a 95% confidence interval for self-sufficiency in year 10 for labour immigrants and refugees compared to the control group of Swedish-born. Crude and adjusted models are presented including age, sex, educational level, civil status and children living at home. We chose to also adjust for age and sex, as there are differences between labour immigrants and refugees in our cohort. 

We further analysed the risk of not being self-sufficient in year 10 among those labour immigrants, refugees and a control group of Swedish-born who were diagnosed with any of the included diagnoses during years 1–5 and those who were not diagnosed in the same period. Swedish-born individuals without a diagnosis during years 1–5 comprised the control group. In the analysis, we present crude and adjusted models, considering age, sex, educational level, civil status and children living at home. Moreover, a sensitivity analysis was carried out, excluding all individuals who were diagnosed in years 6–9.

## 3. Results

### 3.1. Characteristics

About one-third of the population were women, except for labour immigrants, where about one-fifth were women (Table 1). The mean age at the time the residence permit was received was 32.2 years (labour immigrants, 31.5 years; refugees, 32.4 years) for all immigrants. Furthermore, 67.2% of the cohort were young adults, i.e., between 18 and 35 years at the time the residence permit was received in Sweden. Hence, they were at an age where being established in the labour market is crucial to be able to gain self-sufficiency throughout their lifespan. Most refugees were married/in partnership, while the number of those in marriage/in partnership was much lower among labour immigrants and Swedish-born. A high proportion of immigrants were from countries in the Middle East, both among labour immigrants (40.1%) and among refugees (78.3%).

The proportion of individuals with at least one of the included diagnoses during years 1–5 after receiving a residence permit was 17.5% for refugee men and 21.5% for refugee women (Table 2). The corresponding numbers among the control group of Swedish-born were 14.2% among men and 17.7% among women. Labour immigrants had the lowest proportion of individuals diagnosed during the period, with 6.0% among men and 9.6% among women. Among the diagnoses included in this study, the vast majority of the first diagnoses were within the ICD-10 group of psychiatric diseases or diseases of the musculoskeletal system; moreover, this figure did not differ considerably depending on sex or reason for seeking a residence permit. Other anxiety disorders (F41), depressive episodes (F32) and reactions to severe stress and adjustment disorders (F43) constituted 85% of the psychiatric diagnoses in this study; furthermore, this did not differ between labour immigrants, refugees and Swedish-born. The two diagnoses of back pain (M54) and other soft tissue disorders (M79) constituted approximately 70% of the included diagnoses of the musculoskeletal system in all groups, irrespective of the reason for seeking a residence permit or being Swedish-born (numbers not shown). For 13% of the refugees and 16% of the Swedish-born, the first diagnosis was from hospitalisation, while the remaining diagnoses were from physician visits within specialised outpatient care (numbers not shown). Among labour immigrants, there were only three cases of hospitalisation in total (all of them were men). 

### 3.2. Income from Work in the First 10 Years after Receiving a Residence Permit 

**Figure 1:**
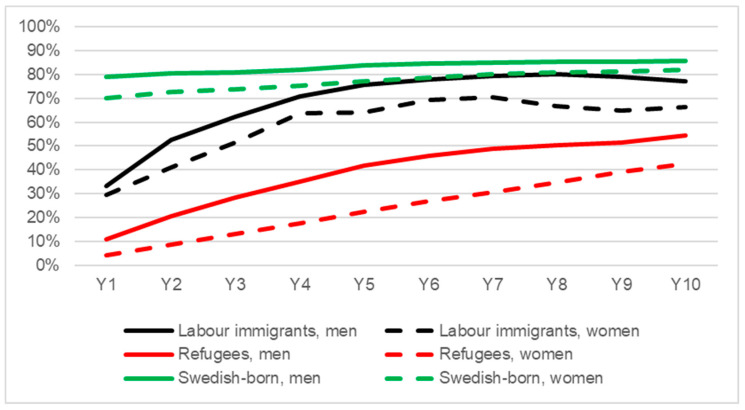
The proportion of labour immigrants, refugees and Swedish-born who have earned over 1.75 PBA ^1^ from paid work (self-sufficiency) 1–10 years after receiving a residence permit ^2^ (stratified by sex, *n* = 169,863). ^1^ Price base amount, an annual figure, e.g., benefits taking into account inflation; ^2^ Year of residence permit = year 0. Swedish-born were given a fictive “year 0” 2000–2006 and were matched by age and sex; approx. 5 Swedish-born to each immigrant.

In all groups, the proportion of self-sufficiency between the first and the last year increased. Refugees had the steepest increase, although from a lower starting point compared to both Swedish-born and labour immigrants (Figure 1). Swedish-born individuals had the highest proportion of self-sufficiency, where about 80% of them were self-sufficient during the 10-year period. Labour immigrants had an approximately ten percentage points lower proportion of self-sufficiency compared to Swedish-born. Throughout the period, refugees had the lowest percentage of self-sufficiency, where about 50% were self-sufficient 10 years after arrival in Sweden. Women within all groups had a slightly smaller proportion of self-sufficiency from work income compared to their male counterparts for the entire study period. The HRs for not reaching self-sufficiency during the tenth year after receiving a residence permit were 1.62 (CI: 1.45–1.81) among labour immigrants and 3.23 (CI: 3.16–3.30) among refugees compared to the control group of Swedish-born in the crude model (Table 3). In the adjusted model, the risk for labour immigrants increased slightly (HR: 1.79 (1.60–2.00)), while it decreased slightly for refugees (HR: 2.68 (2.61–2.75)); however, immigrants still had a substantial higher risk of not being self-sufficient compared to the Swedish-born. 

### 3.3. Sustainable Income

**Figure 2:**
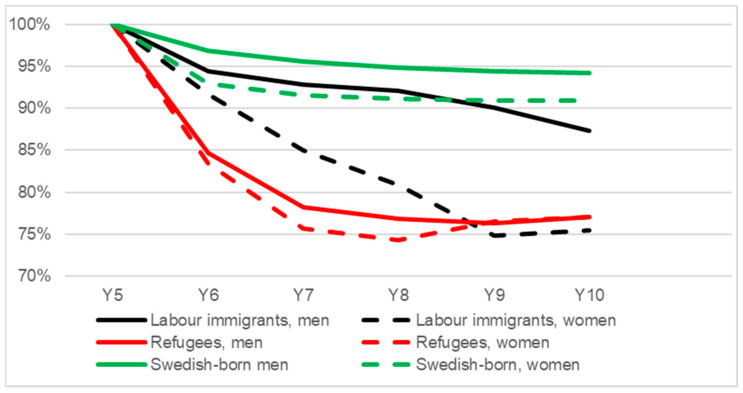
The proportion with an income of 1.75 PBA or more from paid work (self-sufficiency) at year 6–10 for the shares of labour immigrants, refugees and Swedish-born that earned 1.75 PBA ^1^ at year 5 after receiving a residence permit ^2^ (stratified by sex, *n* = 127,133). ^1^ Price base amount, an annual figure, e.g., benefits taking into account inflation; ^2^ Year of residence permit = year 0. Swedish-born were given a fictive “year 0” 2000–2006 and were matched by age and sex; approx. 5 Swedish-born to each immigrant.

When studying the proportion of self-sufficiency in years 6–10 for those who were self-sufficient five years after receiving a residence permit, we found that a larger proportion within the control group of Swedish-born retained their proportion of self-sufficiency during years 6–10 compared to labour immigrants and refugees, in descending order (Figure 2). The proportion of self-sufficiency decreased more among labour immigrants and Swedish-born women compared to their male counterparts, while refugee men and women had similar figures during the period. 

**Figure 3:**
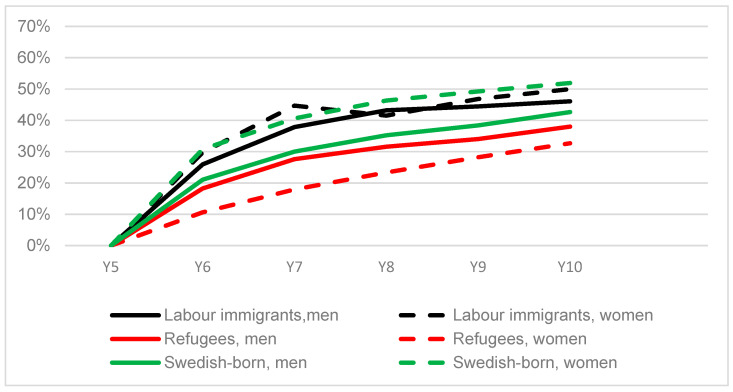
Proportion with an income of 1.75 PBA or more from paid work (self-sufficiency) during years 6–10 for the shares of labour immigrants, refugees and Swedish-born who earned less than 1.75 PBA ^1^ at year 5 after receiving a residence permit ^2^ (stratified by sex, *n* = 42 730). ^1^ Price base amount, an annual figure, e.g., benefits taking into account inflation; ^2^ Year of residence permit = year 0. Swedish-born were given a fictive “year 0” 2000–2006 and were matched by age and sex; approx. 5 Swedish-born to each immigrant.

We also studied the development of self-sufficiency in years 6–10 for those who were not self-sufficient five years after receiving a residence permit (Figure 3). At year 10, the proportion of self-sufficient individuals was 33% among refugee women and 38% among refugee men, which was lower than among labour immigrants and Swedish-born. Among refugees, women had a lower increase in the proportion of self-sufficiency compared to their male counterparts during years 6–10, while Swedish-born women had a higher increase compared to men. Labour immigrant men and women presented a similar development during the period. 

### 3.4. The Impact of Health on Sustainable Earnings

**Figure 4:**
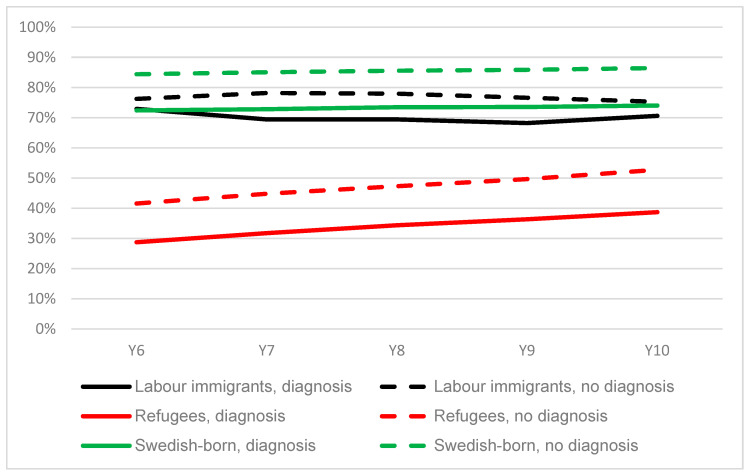
The proportion of labour immigrants, refugees and Swedish-born with or without a diagnosis from hospitalisation or specialised outpatient care ^1^ at years 1–5 with an income of 1.75 PBA or more from paid work during years 6–10 (*n* = 169,863). ^1^ Diagnosis from ICD-10: F31–F38, F41, F43, F45, F48, I10–I13, I20–I25, I50, I60–I69, J13–J18, J20, J40–J45, J60–J69, M05–M06, M15–M17, M50–M54, M65, M70–M71, M77–M79.

Descriptive figures showed that among refugees who had been hospitalised or visited a physician within specialised outpatient care due to any of the included diagnoses during years 1–5 after receiving a residence permit, the proportion of self-sufficiency in year 10 was 39%, compared to 52% among those who were not diagnosed. In both groups, the proportion of self-sufficiency increased during the period; however, the difference between the groups was maintained throughout the period (Figure 4). Among the Swedish-born who had been diagnosed at a hospital or visited a physician within specialised outpatient care during years 1–5, the proportion of self-sufficiency was 74% in year 10, which was about 12 percentage points lower than it was among those who were not diagnosed; moreover, these figures were rather constant during the period. The difference between individuals with or without a diagnosis during years 1–5 was the smallest among the labour immigrants.

Results from the Cox regression analysis showed that a diagnosis from a hospital or a physician visit within specialised outpatient care during years 1–5 after receiving a residence permit increased the risk of not reaching self-sufficiency in year 10 in all groups. Refugees with a diagnosis presented a more than three-fold higher risk of not reaching self-sufficiency in year 10 compared to the Swedish-born with no diagnoses (HR: 3.51 (CI: 3.36–3.67)) (Table 4). The risk of not reaching self-sufficiency among refugees with no diagnosis was also higher than it was among the Swedish-born without a diagnosis ((HR: 2.90 (CI: 2.82–2.99). For labour immigrants with a diagnosis during years 1–5 after receiving a residence permit, the risk of not reaching self-sufficiency in year 10 was doubled compared to Swedish-born with no diagnosis (HR: 2.15 (CI: 1.45–3.18)); moreover, for Swedish-born with a diagnosis, the risk was 70% higher (HR: 1.74 (CI: 1.69–1.79)) compared to Swedish-born with no diagnosis. Excluding all individuals who were not diagnosed at a hospital or did not visit a physician within specialised outpatient care during years 6–9 did not change the results. 

## 4. Discussion

### 4.1. Main Findings

About 50% of the refugees reached self-sufficiency 10 years after receiving a residence permit in Sweden. Corresponding figures were around 75% for labour immigrants and about 80% for Swedish-born. The proportion of refugees who reached self-sufficiency increased rapidly from the first year after receiving a residence permit to five years after in Sweden, then flattened out at around 50%. There was also some dynamic movement in the figures; those with no self-sufficiency during years 1–5 could reach self-sufficiency in years 6–10, and vice versa. Refugees had the highest risk of losing self-sufficiency compared to labour immigrants and Swedish-born. Ten years after residency, the risk of not reaching self-sufficiency was 40% higher among labour immigrants and more than two-fold higher among refugees compared to Swedish-born. Having a diagnosis from specialised health care during the first five years in Sweden affected the ability to gain self-sufficiency through gainful work during years 6–10 in all groups. However, the additional impact of having a diagnosis did not seem to differ much between refugees and Swedish-born individuals, as a diagnosis similarly decreased the possibility of reaching self-sufficiency in all groups.

### 4.2. Self-Sufficiency 

There were substantial differences between labour immigrants and refugees compared to the control group of Swedish-born regarding self-sufficiency during the follow-up period. As expected, the gap was largest at year 1 but diminished rapidly during the five years after receiving a residence permit in Sweden. The proportion of refugees who reached self-sufficiency 10 years after receiving a residence permit was around 50%. This indicates that many refugees seemed to struggle to reach self-sufficiency during their first 10 years in Sweden. Labour immigrants seemed to have an easier time reaching self-sufficiency, probably because they already had work when arriving in Sweden and had lower rates of ill health during the first five years in the country. Labour immigrants had only a five percentage points lower proportion with self-sufficiency compared to Swedish-born, where 80% of the population of working age were self-sufficient. Other studies have also found that immigrants, especially refugees, have difficulties establishing themselves in the labour market [14,15,16,17]. They may be receiving a disability pension, sickness absence, be unemployed, have municipal support, etc. From a societal perspective, there will be a very large loss of production and greater welfare payments if the absence from work becomes permanent. This will also hamper social integration, as having employment has been seen to benefit the integration process. Being outside the labour market will also have a detrimental effect on the individuals themselves, who will be marginalised. Although Sweden is considered to have had the most comprehensive integration policies of all EU countries at the beginning of the 2000s, the gap between Swedish-born and immigrants regarding labour market participation is still wide [27]. A study has found that information and guidance, employment, language skills and culture, and health and well-being are major aspects that influence social and economic integration [28]. Further, as immigrants cannot always make individual choices, employers, colleagues, and other stakeholders must take part in the process to make the integration work [29,30]. 

The proportion of women asylum seekers in Sweden has during the 2000s been around 35–40 per cent [31], and one explanation is that women arrive in Sweden after their spouse, and therefore receive a status as family reunion immigrants [32]. Among labour immigrants, the share of women has during the 2000s been around 25 per cent [33]. Women had a slightly lower rate of self-sufficiency compared to men. Other studies have found that a higher proportion of refugee women seem to be outside the labour market compared to Swedish-born women, but also compared to refugee men [34]. Perhaps our definition of self-sufficiency is set rather low, and this, therefore, may explain why the sex difference was small, especially 10 years after receiving a residence permit. However, there seem to be larger discrepancies earlier in the period, and women seem to struggle more to find a place in the labour market than men [34]. 

Refugees obtaining self-sufficiency during year five had a higher probability of losing self-sufficiency during years 6–10 compared to both labour migrants and the control group of Swedish-born individuals. Refugees also had the lowest probability of gaining self-sufficiency during years 6–10 when not having self-sufficiency in year five. One must consider that due to the healthy migrant effect, refugees who arrive in Sweden are mostly healthier than their counterparts remaining in their home country [35], and also compared to the “most unhealthy” Swedish-born control group. This shows that the vulnerability among refugees in the labour market is high. There is a theoretical concept called the “core–periphery theory”, which means that there is a group in the labour market that has a strong connection to the labour market (core); in contrast, vulnerable groups in the periphery have a weaker connection to the labour market [36]. Those who are vulnerable may be the first who are fired during a recession and the last to be hired in prosperous times. It is evident that refugees may be part of the peripheral labour market, and that measures must be taken to increase the labour market participation among them. 

### 4.3. The Impact of Health on Self-Sufficiency

Having a diagnosis from specialised health care during the first five years in Sweden impacted self-sufficiency in all groups. However, this impact did not seem to differ much between refugees and Swedish-born. The regression analysis showed a considerably higher risk of not reaching self-sufficiency for immigrants, especially refugees, independent of diagnoses compared to the control group of Swedish-born with no diagnosis. Refugees with a diagnosis had the highest risk of all groups of not reaching self-sufficiency in year 10. For labour immigrants, the risk of not reaching self-sufficiency did not differ between those with and those without a diagnosis. One reason may be that the labour immigrants in this cohort suffered from less severe health conditions compared to the other two groups, as there were only three cases of hospitalisation among labour immigrants. 

Being an immigrant, rather than having poor health status, explained a major part of the difficulties in becoming established in the labour market. This is in line with other studies on immigrant and labour market marginalisation [16]. In general populations, studies on the effect of health status on self-sufficiency show that chronic disease may hamper the possibility of having and keeping employment, especially in lower-educated workers [37,38]. One reason for this may be that lower-educated workers are often involved in more physically demanding occupations; another reason may be that they suffer from more severe illnesses [39]. In our cohort, only 17% of refugees had a tertiary education compared to 33% among the Swedish-born. Therefore, it can be expected that a poor health status will affect one’s self-sufficiency more among refugees than among the control group of Swedish-born. However, and somewhat surprisingly, this did not seem to be the case in our study. 

Research on immigrants’ health has shown a deterioration of health with time since immigration, which was also seen in Sweden [23,40]. Reasons behind the deteriorating health in immigrant populations may include factors such as socioeconomic deprivation, adapting to unhealthy behaviours and barriers to receiving adequate health care [41]. Unemployment may also play a role in explaining the deteriorating health of immigrant populations [6,42]. Thus, lack of self-sufficiency and health status may constitute a negative feedback loop [37]. In our cohort, the proportion of refugees with a diagnosis from hospitalisation or specialised outpatient care in the first five years after receiving a residence permit was higher compared to the control group of Swedish-born of the same age and sex. Thus, it is important to increase opportunities for refugees to gain self-sufficiency to have a positive impact on their health and integration. Treatment and rehabilitation to increase labour market opportunities for individuals with a diagnosis are important for all groups, including Swedish-born.

### 4.4. Strengths and Limitations

Major strengths of the data at our disposal are as follows: first, the high quality of the Swedish national registers; second, we can differentiate between immigrants regarding their reason for seeking a residence permit; third, we can follow annual cohorts of all labour immigrants and refugees for 10 years from when they received their residence permit; fourth, the inclusion of a control group of Swedish-born individuals of the same age and sex as the immigrants; fifth, the contrast between the immigrants and the Swedish-born control group, as we only included immigrants with parents not born in Sweden and Swedish-born with two parents born in Sweden. Moreover, by using verified diagnoses from medical records, instead of self-reported data, to classify the health status of the population, we avoided bias due to different perceptions of health between the groups. The coverage of the inpatient register is very high, with a primary diagnosis listed in 99% of all hospital discharges [43]. The coverage in the register of specialised outpatient care is less comprehensive than it is for the inpatient register due to inadequate reporting by some caregivers; furthermore, in about 20% of the cases where a patient visits a physician, no diagnosis is registered [43]. However, as missing data in the outpatient register varies with time and geographic region in Sweden, and as our data were collected during an extended period and from different regions, there is no reason to believe that systematic differences between labour immigrants, refugees and the control group of Swedish-born would have biased the results. Some limitations also need to be mentioned. In our study, we were not able to assess the impact of less severe diseases on self-sustainability, because Sweden lacks a register of diagnoses in primary care. Consequently, the impact of the health status on workability for immigrants and for the control group of Swedish-born could be similar because we only included the relatively severe conditions referred to specialised health care. Another limitation was that we could not establish the extent to which the increased risk of not being self-sufficient in year 10 among individuals with a diagnosis during years 1–5 was due to lost opportunities in the labour market in the years following diagnosis or due to ongoing health impairment. However, the sensitivity analyses, where individuals with a diagnosis from hospitalisation or physician visits within specialised outpatient care during years 6–9 were excluded, did not change the results regarding the relationship between a diagnosis in years 1–5 and self-sufficiency in year 10. 

## 5. Conclusions 

About 50% of non-European refugees in Sweden reached economic self-sufficiency after 10 years in Sweden compared to 80% among the corresponding control group of Swedish-born. Moreover, a higher proportion of refugees had difficulties in keeping self-sufficiency during the studied period. Having a diagnosis from the hospital or specialised outpatient health care during the first five years in Sweden had an impact on self-sufficiency in all groups; however, the impact of having a diagnosis did not seem to differ between refugees and Swedish-born. Thus, immigration status, rather than poor health, may explain immigrants’ difficulties in becoming established in the labour market. Future research should therefore focus on factors promoting a faster establishment in the labour market, as well as the long-term sustainability of labour market integration. 

## Figures and Tables

**Table 1:** Characteristics of the study population at year 1 after receiving a residence permit (*n* = 169,863).

	Labour Immigrants	Refugees	Swedish-Born	Total
	*n* (%)	*n* (%)	*n* (%)	*n* (%)
**Sex**				
Men	998 (79.3)	15,349 (64.3)	96,708 (66.8)	113,055 (66.6)
Women	261 (20.7)	8510 (35.7)	48,037 (33.2)	56,808 (33.4)
**Age**				
18–25 years	510 (40.5)	6255 (26.2)	37,682 (26.0)	44,447 (26.2)
26–35 years	558 (44.3)	9566 (40.1)	60,342 (41.7)	70,466 (41.5)
36–45 years	146 (11.6)	5820 (24.4)	34,245 (23.7)	40,211 (23.7)
46–55 years	45 (3.6)	2218 (9.3)	12,476 (8.6)	14,739 (8.7)
**Educational level**				
Primary school	118 (9.4)	8764 (36.7)	13,759 (13.2)	22,641 (13.3)
Secondary school	114 (9.1)	7011 (29.4)	70,238 (48.5)	77,363 (45.6)
Tertiary education	970 (77.1)	7436 (31.2)	60,237 (41.6)	68,643 (40.4)
Missing	57 (4.5)	648 (2.7)	511 (0.4)	1216 (0.7)
**Family status**				
Married/Partnership	400 (31.8)	14,020 (58.8)	37,149 (25.7)	51,569 (30.4)
Other	859 (68.2)	9839 (41.2)	107,596 (74.3)	118,294 (69.6)
**Children < 18 home**				
Yes	133 (10.6)	8678 (36.4)	79,295 (54.8)	88,106 (51.9)
No	1126 (89.4)	15,181 (63.6)	65,450 (45.2)	81,757 (48.1)
**Country of origin**				
The Middle East	505 (40.1)	18,687 (78.3)	**-**	-
Northeast Africa	36 (2.9)	3946 (15.5)	**-**	-
Central and South America	131 (10.4)	783 (3.3)	**-**	-
East Asia	587 (46.6)	443 (1.9)	**-**	-
**Total (% of cohort)**	**1259 (0.7)**	**23,859 (14.0)**	**144,745 (85.2)**	**169,863 (100)**

**Table 2:** Number and proportion of individuals with the first diagnosis from hospitalisation or specialised outpatient health care 1–5 years after residency, presented per diagnosis group and stratified by reason for seeking a residence permit and sex (*n* = 169,863).

	Labour Immigrants		Refugees		Swedish-Born	
	Women *n* (%)	Men *n* (%)	Women *n* (%)	Men *n* (%)	Women *n* (%)	Men *n* (%)
Psychiatric ^1^	9 (3.5)	21 (2.1)	824 (9.7)	934 (6.1)	2744 (5.7)	3310 (3.4)
Cardiovascular ^2^	0	2 (0.2)	82 (1.0)	229 (1.5)	379 (0.8)	925 (1.0)
Respiratory ^3^	5 (1.9)	3 (0.3)	133 (1.6)	222 (1.5)	1134 (2.4)	1826 (1.9)
Musculoskeletal ^4^	11 (4.2)	34 (3.4)	793 (9.3)	1302 (8.5)	4229 (8.8)	7658 (7.9)
Any diagnosis ^5^	25 (9.6)	60 (6.0)	1832 (21.5)	2687 (17.5)	8486 (17.7)	13,719 (14.2)

^1^ ICD-10: F31–F38, F41, F43, F45, F48; ^2^ ICD-10: I10–I13, I20–I25, I50, I60–I69; ^3^ ICD-10: J13–J18, J20, J40–J45, J60–J69, J92; ^4^ ICD-10: M05–M06, M15–M17, M50–M54, M65, M70–M71, M77–M79; ^5^ Any of the above-mentioned diagnoses.

**Table 3:** Hazard ratios (HRs) with 95% confidence intervals (CIs) for the risk of not reaching self-sufficiency 10 years after immigration among labour immigrants and refugees compared to Swedish-born of the same age.

	Crude	Adjusted ^1^
	HR (95% CI)	
Swedish-born	1	1
Labour immigrant	1.62 (1.45–1.81)	1.79 (1.60–2.00)
Refugees	3.23 (3.16–3.30)	2.68 (2.61–2.75)

^1^ Adjusted for sex, age, educational level, family status and children living at home.

**Table 4:** Hazard ratios (HRs) with 95% confidence intervals (CIs) for the risk of not reaching self-sufficiency 10 years after immigration among labour immigrants and refugees with or without a diagnosis from hospitalisation or specialised outpatient care ^1^ compared to Swedish-born without a diagnosis.

	Crude	Adjusted ^2^
	HR (95% CI)	
Swedish-born with no diagnosis	1	1
Swedish-born with a diagnosis	1.92 (1.86–1.97)	1.74 (1.69–1.79)
Labour immigrants with no diagnosis	1.82 (1.62–2.05)	1.96 (1.75–2.21)
Labour immigrants with a diagnosis	2.17 (1.47–3.21)	2.15 (1.45–3.18)
Refugees with no diagnosis	3.48 (3.40–3.57)	2.90 (2.82–2.99)
Refugees with a diagnosis	4.52 (4.34–4.71)	3.51 (3.36–3.67)

^1^ Diagnosis from ICD-10: F31–F38, F41, F43, F45, F48, I10–I13, I20–I25, I50, I60–I69, J13–J18, J20, J40–J45, J60–J69, M05–M06, M15–M17, M50–M54, M65, M70–M71, M77–M79. ^2^ Adjusted for sex, age, educational level, family status and children living at home.

## Data Availability

The data that support the findings of this study are available from Statistics Sweden and the Swedish National Board of Health and Welfare. However, restrictions apply to the availability of these data on an individual level and they can therefore not be made publicly available.
